# Associations of serum creatinine and blood urea nitrogen with depressive symptoms among community-dwelling older adults in Guangzhou, China: a cross-sectional study

**DOI:** 10.3389/fpsyt.2026.1746646

**Published:** 2026-02-09

**Authors:** Jinping Huang, Yuanzheng Fu, Yushui Fu, Yangjian Pan, Yurong Hu, Jinquan Zhang, Wanwei Guo, Xiaoyan Du

**Affiliations:** 1 Department of Dermatology, the Affiliated Guangdong Second Provincial General Hospital of Jinan University, Guangzhou, China; 2 Department of Science and Education, the Affiliated Guangdong Second Provincial General Hospital of Jinan University, Guangzhou, China; 3 Department of Neonatology, Hainan Women and Children’s Medical Center, Haikou, China; 4 General Practice Department, The Affiliated Guangdong Second Provincial General Hospital of Jinan University, Guangzhou, China; 5 General Outpatient Clinic, Dashi Panyu Community Health Service Center, Guangzhou, China

**Keywords:** blood urea nitrogen, community-based study, depressive symptoms, EGFR, older adults, renal function, restricted cubic splines, serum creatinine

## Abstract

**Background:**

Depression is a common and serious mental health concern among older adults, with depressive symptoms highly prevalent in elderly populations across China. Although several studies have examined the associations between renal biomarkers—such as serum creatinine (Scr) and blood urea nitrogen (BUN)—and depressive symptoms, evidence remains limited, particularly in community-dwelling older adults. This study aimed to investigate the associations of renal biomarkers (Scr, BUN, and their ratio) with depressive symptoms in a community-based cohort of older adults in Guangzhou, China.

**Methods:**

This cross-sectional study included 1, 994 adults aged ≥ 65 years from community-based health screenings in Guangzhou. Depressive symptoms were assessed using the Patient Health Questionnaire-9 (PHQ-9). Renal function was evaluated using Scr, BUN, and the BUN/Scr ratio. Multivariable logistic regression models were used to assess the association between these renal biomarkers and depressive symptoms, adjusting for potential confounders including age, sex, body mass index (BMI), and chronic disease history. To ensure robustness, we conducted sensitivity analyses by substituting Scr with estimated glomerular filtration rate (eGFR). Restricted cubic splines were used to evaluate dose-response shapes. Subgroup analyses tested for effect modification by sex, age group, BMI category, and histories of hypertension, diabetes, and dyslipidemia.

**Results:**

Among the 1, 994 participants (median age: 71 years), 158 (7.9%) exhibited depressive symptoms. After full adjustment, higher Scr (adjusted OR per 1 μmol/L = 1.002, 95% CI 1.000–1.004) and BUN (adjusted OR per 1 mmol/L = 1.064, 95% CI 1.007–1.123) were independently associated with higher odds of depressive symptoms, whereas the BUN/Scr ratio showed no significant association. In sensitivity analyses, eGFR was inversely associated with depressive symptoms (OR = 0.992, 95% CI 0.985–0.999). Restricted cubic spline analyses revealed an approximately linear dose–response for BUN (P for nonlinearity = 0.407). Subgroup analyses indicated significant effect modification for Scr, with stronger associations observed among participants aged ≥70 years, those with overweight/obesity (BMI ≥24 kg/m²), and those with hypertension (all P for interaction< 0.05).

**Conclusions:**

Higher Scr and BUN levels were independently associated with increased odds of depressive symptoms among community-dwelling older adults. The association of Scr with depression was particularly pronounced in older individuals, those with higher BMI, and those with hypertension.

## Introduction

Depression is a leading cause of disability worldwide and disproportionately affects older adults (1). In China—one of the world’s most rapidly ageing societies—surveillance data indicate that the prevalence of depressive symptoms among older adults increased markedly between 2013 and 2020, underscoring the need for scalable, community-level strategies for early detection and prevention (2). Regional evidence from Guangdong Province likewise shows a substantial prevalence of depressive symptoms among community-dwelling older adults, and comparative syntheses of pre- and mid-pandemic data suggest meaningful temporal variation in estimates (3, 4).

Beyond psychosocial determinants, kidney health has emerged as a biologically plausible correlate of late-life mental health. Meta-analytic evidence indicates that depression is highly prevalent across all stages of chronic kidney disease (CKD) (5). Importantly, large-scale observational cohorts suggest a bidirectional relationship: kidney impairment is associated with an increased risk of subsequent depression requiring treatment, whereas depressive symptoms themselves may precede and predict incident CKD (6–8). Contemporary mechanistic and clinical reviews implicate a uremic milieu, microvascular injury, chronic inflammation, oxidative stress, and broader brain–kidney network dysfunction as converging pathways linking renal impairment to affective disturbances, particularly depression, in ageing populations (9, 10). Extending these established mechanisms, recent advances in kidney–brain crosstalk research highlight uremic toxin–induced neuroinflammation—including aryl hydrocarbon receptor (AhR)-related signaling—as a key driver of cerebral dysfunction, offering a biologically plausible explanation for its role in mood disorders (11, 12).

At the community level, routine biochemical indicators may reflect clinically relevant links between kidney function and mood; however, findings remain inconsistent across studies and populations, underscoring the need for further evaluation in older adults (13). In a nationwide analysis, higher blood urea nitrogen (BUN) levels were associated with an increased risk of depression, with the pattern varying by type 2 diabetes status (14). Conversely, a large ageing cohort reported that lower serum creatinine (Scr)—a biomarker influenced by both renal function and muscle mass—was linked to a higher risk of depression, suggesting possible nonlinearity and population heterogeneity (15, 16). The BUN-to-creatinine ratio (BUN/Scr) has also been related to cognitive function, with depressive symptoms acting as a mediator, implying that mood may lie along the pathway between renal biochemistry and neurocognitive outcomes (17). Additional evidence indicates sex-specific associations when renal function is represented by eGFR dynamics (18). Despite these advances, evidence among Chinese community-dwelling adults aged ≥ 65 years remains scarce and inconsistent, particularly regarding routinely measured biomarkers (Scr, BUN) and their ratio (BUN/Scr), and few studies in this context have formally examined potential dose–response nonlinearity.

Against this background, we analyzed a community-based cohort of adults aged ≥65 years in Guangzhou, China, to examine the associations of Scr, BUN, and BUN/Scr with depressive symptoms assessed using the Patient Health Questionnaire-9 (PHQ-9). Multivariable logistic regression models were employed to estimate these associations, and restricted cubic splines were used to characterize potential dose–response relationships. Subgroup analyses were conducted by sex, age group, body mass index (BMI) category, and histories of hypertension, diabetes, and dyslipidemia. As a sensitivity analysis, Scr was replaced with the creatinine-based estimated glomerular filtration rate (eGFR) to evaluate the robustness of findings across biomarker metrics. Given that Scr and BUN are low-cost and widely available laboratory tests routinely incorporated into health check-ups, elucidating their relationships with late-life depressive symptoms may help inform feasible strategies for risk stratification, early detection, and follow-up in community and primary care settings.

## Methods

### Study population

This study recruited adults aged ≥65 years who underwent community health examinations at a community hospital in Guangzhou between January and December 2024. A total of 1, 994 participants were included. The inclusion criteria were as follows: (1) residence in the study area at the time of recruitment; (2) age ≥ 65 years; and (3) participants or their family members understood the study objectives and procedures and voluntarily agreed to participate. Exclusion criteria were as follows: (1) refusal to cooperate with the survey; or (2) inability to participate due to physical limitations.

### Assessment of exposure

Fasting venous blood samples were collected from all participants, and serum was separated for analysis. BUN and Scr levels were measured using enzymatic assays with reagents from Mindray (China). All analyses were conducted on a Mindray BS-600 automated biochemical analyzer in accordance with the manufacturer’s standard operating procedures.

The BUN-to-creatinine ratio (BUN/Scr) was calculated as (19):


BUN/Scr=[BUN(mmol/L)×2.8×88.4]÷Scr (μmol/L)

The estimated glomerular filtration rate (eGFR) was calculated as (20):


$$\text{eGFR}=175\times {\left[\text{Scr }\left(\mu \text{mol}/\text{L}\right)/88.4\right]}^{-1.234}\times {\left(\text{age}\right)}^{-0.179}\times 0.79\;\left(\text{if female}\right)$$

All reagents and assay kits were provided by Mindray Co., Ltd. (China), and all biochemical measurements were performed by Guangzhou KingMed Diagnostics Group Co., Ltd.

### Outcome ascertainment

Depressive symptoms were evaluated using the Patient Health Questionnaire-9 (PHQ-9), a widely used self-report instrument for screening depression across diverse populations (21). The PHQ-9 is simple, convenient, and has demonstrated high reliability and validity. It comprises nine items, each rated on a four-point scale from 0 (“not at all”) to 3 (“nearly every day”), yielding a total score ranging from 0 to 27. Higher total scores indicate greater symptom severity. According to standard thresholds, scores of 0–4 indicate no depression, 5–9 mild depression, 10–14 moderate depression, 15–19 moderately severe depression, and ≥ 20 severe depression. In this study, a PHQ-9 score ≥ 5 was used to define the presence of depressive symptoms.

### Covariates

Information on demographic characteristics (age, sex, marital status, education level, and occupation), health status (history of hypertension, dyslipidemia, and family history of diabetes), and lifestyle behaviours (smoking, alcohol consumption, and physical activity) was obtained through structured questionnaires. BMI was calculated as weight (kg) divided by height squared (m²) and classified according to the Chinese adult criteria as follows: underweight (< 18.5 kg/m²), normal weight (18.5–23.9 kg/m²), overweight (24.0–27.9 kg/m²), and obese (≥ 28.0 kg/m²) (22, 23).

### Data analysis

Descriptive statistics were used to summarize the characteristics of the participants. Categorical variables were expressed as counts (percentages) and compared between groups using the χ² test. Because serum biomarkers exhibited right-skewed distributions, continuous variables were expressed as medians (interquartile range, IQR) and compared using the Wilcoxon rank-sum test. Depressive symptoms were defined *a priori* as PHQ-9 scores ≥5 (binary outcome).

For association analyses, we fitted multivariable logistic regression models and reported odds ratios (ORs) with 95% confidence intervals (CIs). Each renal biomarker—blood urea nitrogen (BUN, mmol/L), serum creatinine (Scr, μmol/L), and the BUN-to-creatinine ratio (BUN/Scr, unitless)—was entered separately as a continuous predictor. The BUN/Scr ratio was calculated as BUN/Scr = [BUN (mmol/L) × 2.8 × 88.4] ÷ Scr (μmol/L), where 2.8 and 88.4 are the conventional SI-to-US conversion factors for BUN and Scr, respectively (19). Effect sizes were reported on their native measurement scales (i.e., per 1 mmol/L increase in BUN and per 1 μmol/L increase in Scr).

Four hierarchical models were constructed as follows:

Model 0: unadjusted;
Model 1: adjusted for age, sex, and body mass index (BMI);
Model 2: Model 1 plus marital status, education, occupation, smoking, and alcohol consumption;
Model 3 (fully adjusted): Model 2 plus histories of hypertension, diabetes, dyslipidemia, and exercise frequency.

To explore potential non-linear dose–response relationships, restricted cubic spline (RCS) models were fitted with three knots placed at the 10th, 50th, and 90th percentiles of each biomarker’s distribution, using the median as the reference point. Departure from linearity was assessed using Wald tests for the joint significance of the spline terms. The RCS curves present adjusted ORs and 95% CIs derived from Model 3.

Subgroup analyses were prespecified by sex (male/female), age group (65–69, 70–74, 75–79, ≥80 years), BMI category (underweight<18.5 kg/m²; normal 18.5–23.9 kg/m²; overweight 24.0–27.9 kg/m²; obesity ≥28.0 kg/m², Chinese criteria), and histories of hypertension, diabetes, and dyslipidemia (yes/no). Within each stratum, Model 3 was refitted. Effect modification was evaluated on the multiplicative scale by adding cross-product terms (biomarker × subgroup) to Model 3, and Wald tests were used to determine P values for interaction.

For the sensitivity analysis, we assessed the robustness of our findings and reduced potential collinearity with Scr by repeating the primary analyses across all model tiers (Models 0–3), substituting eGFR for Scr. Scr and eGFR were not included in the same model. Results for Model 3 are presented in the main text, with directionally consistent findings across the other models.

All statistical tests were two-sided, with α =0.05. No adjustments for multiple testing were applied to secondary or exploratory analyses. All analyses were conducted using R version 4.3.3, and restricted cubic spline models were fitted with the rms package.

## Results

### Baseline characteristics

Among the 1, 994 participants, the median (Q1, Q3) age was 71 (68, 75) years, and 932 (46.7%) were men. In the total population, the median (Q1, Q3) values were 6.0 (5.0, 7.4) mmol/L for BUN, 81.0 (68.3, 100.0) μmol/L for Scr, 18.0 (14.9, 21.4) for the BUN/Scr ratio, and 80.3 (63.1, 95.3) mL/min/1.73 m² for eGFR. Overall, 158 participants (7.9%; 158/1, 994) had PHQ-9 scores ≥ 5; among these, the median PHQ-9 score was 7 (5, 10). Participants with depressive symptoms differed significantly from those without in marital status, body mass index, self-rated health, self-rated ability of daily living, exercise frequency, and histories of diabetes and hypertension (all P< 0.05; **Table 1**).

**Table 1:** Baseline characteristics of community-dwelling adults aged ≥65 years with and without depressive symptoms.

Characteristic	Depressive (n=158)	Non-depressive group (n=1836)	*χ*^2^/*Z* value	P value
Sex				
Male	66 (41.8)	866 (47.2)	1.492	0.222
Female	92 (58.2)	970 (52.8)		
Age (years)				
65–69	64 (40.5)	742 (40.4)	5.751	0.124
70–74	38 (24.1)	583 (31.8)		
75–79	32 (20.3)	288 (15.7)		
≥80	24 (15.2)	223 (12.1)		
Residence status				
Non-local resident	53 (33.5)	534 (29.1)	1.186	0.276
Local resident	105 (66.5)	1302 (70.9)		
Educational attainment				
Primary school or below	65 (41.1)	793 (43.2)	0.257	0.879
Middle/high school	77 (48.7)	860 (46.8)		
College or above	16 (10.1)	183 (10.0)		
Occupational type				
Primarily non-manual work	8 (5.1)	91 (5.0)	2.100	0.350
Primarily manual work	95 (60.1)	1205 (65.6)		
Other	55 (34.8)	540 (29.4)		
Marital status				
Married	140 (88.6)	1655 (90.1)	12.982	0.005
Never married	3 (1.9)	75 (4.1)		
Divorced	5 (3.2)	12 (0.7)		
Widowed	10 (6.3)	94 (5.1)		
BMI				
Underweight	11 (7.0)	60 (3.3)	18.688	<0.001
Normal weight	64 (40.5)	828 (45.1)		
Overweight	42 (26.6)	657 (35.8)		
Obesity	41 (25.9)	291 (15.8)		
Self-rated health status				
Unsatisfied	36 (22.8)	289 (15.7)	4.788	0.029
Satisfied	122 (77.2)	1547 (84.3)		
Activities of daily living				
Dependent	37 (23.4)	283 (15.4)	6.336	0.012
Independent	121 (76.6)	1553 (84.6)		
Exercise				
No	86 (54.4)	778 (42.4)	8.127	0.004
Yes	72 (45.6)	1058 (57.6)		
Smoking				
Non-smoking	116 (73.4)	1368 (74.5)	0.043	0.836
Smoking	42 (26.6)	468 (25.5)		
Alcohol consumption				
Non-drinking	111 (70.3)	1421 (77.4)	3.779	0.052
Drinking	47 (29.7)	415 (22.6)		
Dental caries				
No	92 (58.2)	1124 (61.2)	0.429	0.513
Yes	66 (41.8)	712 (38.8)		
History of diabetes				
No	114 (72.2)	1498 (81.6)	7.770	0.005
Yes	44 (27.8)	338 (18.4)		
History of hypertension				
No	65 (41.1)	932 (50.8)	5.011	0.025
Yes	93 (58.9)	904 (49.2)		
History of dyslipidemia				
No	62 (39.2)	873 (47.5)	3.706	0.054
Yes	96 (60.8)	963 (52.5)		
BUN [mmol/L, *M* (*Q*_1_, *Q*_3_)]	6.2 (5.1, 7.8)	6.0 (5.0, 7.4)	-1.587	0.056
Scr [μmol/L, *M* (*Q*_1_, *Q*_3_)]	85.0 (70.0, 114.3)	81.0 (68.0, 99.0)	-1.556	0.060
BUN/Scr	17.9 (14.4, 21.0)	18.0 (14.9, 21.4)	-0.339	0.367
eGFR [mL/min/1.73 m^2^, *M* (*Q*_1_, *Q*_3_)]	76 (53.4, 91.5)	80.8 (63.8, 95.6)	-2.320	0.010

BMI, body mass index; BUN, blood urea nitrogen; Scr, serum creatinine; BUN/Scr, BUN-to-creatinine ratio. The BUN/Scr ratio was calculated after unit harmonization as [BUN (mmol/L) × 2.8 × 88.4] ÷ Scr (μmol/L). Estimated glomerular filtration rate (eGFR) was computed as 175 × [Scr (μmol/L)/88.4]^−1.234^ × (age)^−0.179^ × 0.79 (if female).

### Associations of BUN, Scr, and the BUN/Scr ratio with depressive symptoms

In multivariable logistic regression models (**Table 2**), higher BUN levels were consistently associated with greater odds of depressive symptoms across all adjustment models (Model 3: OR = 1.064, 95% CI 1.007–1.123). Scr also showed a modest positive association (Model 3: OR = 1.002, 95% CI 1.000–1.004; P = 0.030); when expressed per 10 μmol/L increase, the OR was approximately 1.02. In contrast, BUN/Scr was not significantly associated with depressive symptoms (Model 3: OR = 0.976, 95% CI 0.942–1.011). In sensitivity analyses—conducted separately from Scr to avoid collinearity—the estimated glomerular filtration rate (eGFR) was inversely associated with depressive symptoms (Model 3: OR = 0.992, 95% CI 0.985–0.999), with directionally consistent results across the other model tiers (Models 0–2).

**Table 2:** Associations of BUN, Scr, and the BUN/Scr ratio with depressive symptoms among community-dwelling adults aged ≥65 years.

Variable	Model 0		Model 1		Model 2		Model 3	
	OR (95% CI)	P	OR (95% CI)	P	OR (95% CI)	P	OR (95% CI)	P
BUN	1.079 (1.033, 1.128)	0.001	1.061 (1.012, 1.112)	0.014	1.065 (1.009, 1.123)	0.022	1.064 (1.007, 1.123)	0.027
Scr	1.002 (1.001, 1.004)	0.002	1.002 (1.000, 1.004)	0.036	1.002 (1.000, 1.004)	0.038	1.002 (1.000, 1.004)	0.027
BUN/Scr	0.986 (0.956, 1.017)	0.368	0.982 (0.950, 1.016)	0.295	0.981 (0.948, 1.015)	0.264	0.976 (0.942, 1.010)	0.167
eGFR^a^	0.991 (0.985, 0.997)	0.002	0.992 (0.986, 0.999)	0.018	0.992 (0.985, 0.999)	0.026	0.992 (0.985, 0.999)	0.025

OR, odds ratio; CI, confidence interval; BUN, blood urea nitrogen (mmol/L); Scr, serum creatinine (μmol/L); BUN/Scr, BUN-to-creatinine ratio (unitless); eGFR, estimated glomerular filtration rate (mL/min/1.73 m²).

Model 0, unadjusted; Model 1, adjusted for age, sex, and BMI; Model 2, additionally adjusted for marital status, educational attainment, occupation, smoking, and alcohol consumption; Model 3, further adjusted for histories of hypertension, diabetes, dyslipidemia, and exercise frequency.

a eGFR was modeled separately (i.e., replacing Scr) to avoid collinearity.

**Figure 1:**
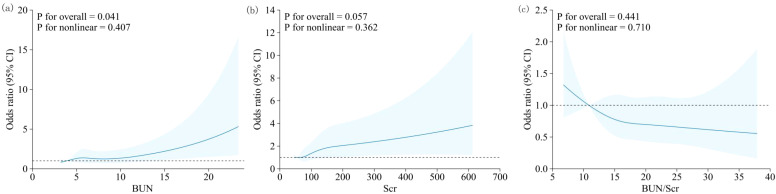
Restricted cubic spline curves for the associations of BUN, Scr, and the BUN/Scr ratio with the risk of depressive symptoms among community-dwelling adults aged ≥65 years. Models were adjusted for age, sex, BMI, marital status, educational attainment, occupation, smoking, alcohol consumption, histories of hypertension, diabetes, and dyslipidemia, and exercise frequency. Abbreviations: BUN, blood urea nitrogen; Scr, serum creatinine; BUN/Scr, BUN-to-creatinine ratio.

Restricted cubic spline analyses (**Figure 1**) revealed an approximately linear increase in the odds of depressive symptoms with higher BUN levels (P for overall = 0.041; P for nonlinearity = 0.407) and suggested a borderline positive association for Scr, with no evidence of nonlinearity (P for overall = 0.057; P for nonlinearity = 0.362). No significant association was observed for the BUN/Scr ratio (P for overall = 0.441; P for nonlinearity = 0.710). The confidence intervals widened at the extremes of the distributions, indicating greater uncertainty in those regions.

**Figure 2:**
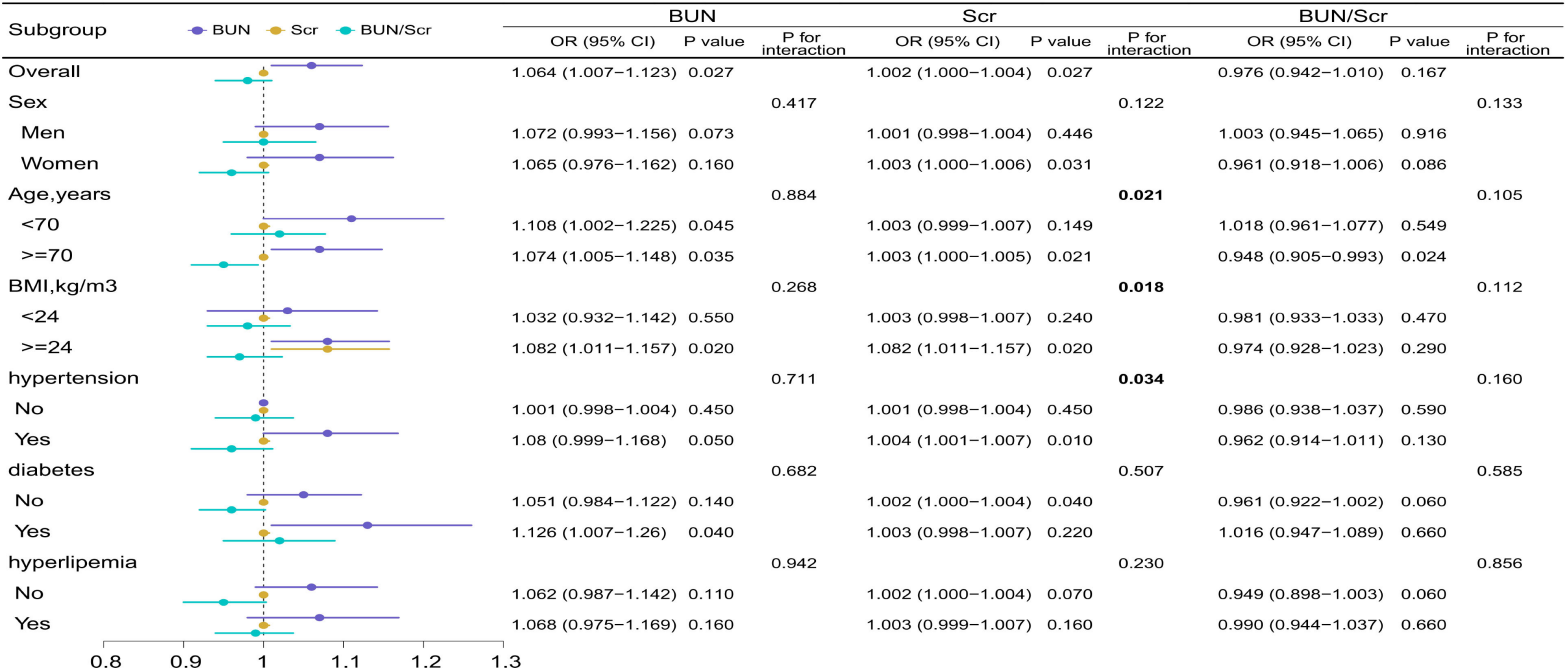
Subgroup analysis of the associations between BUN, Scr, and the BUN/Scr ratio and depressive symptoms among community-dwelling adults aged ≥65 years. Models were adjusted for age, sex, BMI, marital status, educational attainment, occupation, smoking, alcohol consumption, histories of hypertension, diabetes, and dyslipidemia, and exercise frequency. Abbreviations: BUN, blood urea nitrogen; Scr, serum creatinine; BUN/Scr, BUN-to-creatinine ratio.

Subgroup analyses visualized in forest plots (**Figure 2**) showed no meaningful effect modification for BUN (all P for interaction > 0.26). For Scr, stronger associations were observed among participants aged ≥ 70 years (P for interaction = 0.021), those with BMI ≥ 24 kg/m² (P for interaction = 0.018), and those with hypertension (P for interaction = 0.034). No significant interactions were detected for the BUN/Scr ratio (all P for interaction > 0.10). Overall, renal function biomarkers—particularly BUN and Scr—were positively associated with depressive symptoms, whereas BUN/Scr showed null associations, and eGFR demonstrated consistent inverse relationships in sensitivity analyses.

## Discussion

In this community-based sample of adults aged ≥65 years in Guangzhou (n = 1, 994), higher Scr and blood BUN levels were independently associated with increased odds of depressive symptoms after multivariable adjustment, whereas the BUN/Scr ratio showed no clear association. On the original measurement scales, the adjusted odds ratios (ORs) were 1.002 per 1 μmol/L increase in Scr and 1.064 per 1 mmol/L increase in BUN. Restricted cubic spline analyses demonstrated approximately linear associations across the observed ranges; uncertainty widened at the upper extremes where data were sparse, while confidence bands were narrower in regions with denser observations. In a sensitivity analysis substituting Scr with creatinine-based eGFR, higher eGFR was associated with lower odds of depressive symptoms (model 3: OR = 0.992; 95% CI = 0.985–0.999), consistent with the expected inverse relationship between Scr and eGFR, thereby supporting internal consistency.

These findings contribute community-based evidence from China to the broader literature linking kidney health with late-life depressive symptoms. Longitudinal cohort studies have demonstrated that chronic kidney disease (CKD) is associated with an increased risk of incident or treatment-requiring depression, while depressive symptoms themselves may precede and predict accelerated kidney function decline or new-onset CKD (6, 7, 24, 25). Moreover, synthesis studies indicate that depression is highly prevalent among individuals with CKD and is associated with poorer quality of life and greater healthcare utilization (5, 26, 27).

At the biomarker level, the positive association observed for BUN is consistent with nationwide evidence linking elevated urea nitrogen to higher depression risk, with some studies suggesting modification by type 2 diabetes—highlighting the role of metabolic context as a potential source of heterogeneity (14). In contrast, several ageing cohorts have reported an inverse association between serum creatinine and depression risk, often interpreted as reflecting lower muscle mass or nutritional deficiency rather than better renal function per se (15, 28). Variations in body composition, hydration status, dietary patterns, comorbidity profiles (notably diabetes and hypertension), laboratory calibration, and covariate adjustment strategies likely contribute to the divergent findings across studies. Additional evidence indicates that renal function indexed by eGFR may exhibit sex-specific associations with depressive symptoms, and that creatinine- and cystatin C–based estimates can differ substantially, adding further complexity to interpretation (16, 18). From a public health perspective, national and provincial data—including those from Guangdong—continue to document a substantial burden of late-life depressive symptoms, with temporal fluctuations observed around the COVID-19 period (4, 29–33). Utilizing routinely collected biochemical data from community health screenings could therefore support opportunistic depression screening or more targeted case identification in older populations.

Depression was assessed using the Patient Health Questionnaire-9 (PHQ-9), which has been contemporarily validated in Chinese and older adult populations; however, potential age-related differences in reliability and measurement non-invariance warrant cautious interpretation (34–36). Endorsement of somatic symptoms, cognitive status, and cultural expression of mood may influence item responses and, consequently, the estimated associations. Single-time laboratory measurements are also susceptible to short-term biological variability (e.g., hydration status, intercurrent illness, diurnal fluctuations), which can attenuate associations and increase uncertainty. Repeated assessments of both symptoms and biomarkers would help quantify stability and reduce measurement error.

Multiple converging mechanisms may link renal status to affective outcomes in ageing, including a uremic milieu that alters neurotransmission, microvascular injury impairing cerebral perfusion, chronic inflammation and oxidative stress, and broader brain–kidney axis alterations (10, 37). Uremic neurotoxicity can disrupt the blood–brain barrier and compromise neural integrity, with partial reversibility observed after improved toxin clearance or renal replacement therapy (10). Experimental evidence further demonstrates that elevated urea can impair synaptic plasticity and induce depression-like behaviors through carbamylation-mediated suppression of mTOR signalling, while disruptions in urea transport may lead to brain urea accumulation and depressive phenotypes (37, 38). These pathways provide plausible biological explanations for the observed association with BUN and suggest that filtration-independent processes may contribute to mood disturbances in late life. Supporting this, meta-analytic evidence on anti-inflammatory interventions among older adults strengthens the hypothesis of an inflammation-mediated pathway linking renal dysfunction and depression (39).

This study has several methodological and practical strengths. It was conducted in a relatively large, community-based cohort of older Chinese adults, with standardized collection of fasting serum samples and validated assessment of depressive symptoms. The emphasis on routinely available renal biomarkers—Scr, BUN, and the BUN/Scr ratio—adds pragmatic relevance for primary care and community screening, where such indices are already part of routine health check-ups. The prespecified multivariable modelling strategy, which incorporated restricted cubic splines, subgroup analyses, and a substitution-based sensitivity analysis using eGFR, strengthened both the robustness and interpretability of the findings. Moreover, the direction and magnitude of associations remained generally consistent across alternative model specifications and population subgroups (age, sex, BMI, and hypertension), supporting the internal validity of our results and their potential value for risk stratification in late-life depression prevention.

Despite the strengths of this study, several limitations should be acknowledged. First, the cross-sectional design precludes causal inference and warrants caution when interpreting temporal relationships; therefore, the possibility of reverse causation cannot be ruled out. Moreover, our multivariate regression approach, while identifying independent associations, does not allow us to disentangle the potential complex pathways and intermediary mechanisms through which renal markers may influence depressive symptoms. Second, renal biomarkers and PHQ-9 scores were assessed at a single time point, which may introduce short-term biological variability. However, such non-differential measurement error typically biases associations toward the null rather than exaggerating them. Third, although we adjusted for multiple confounders, residual confounding may persist due to unmeasured factors such as dietary intake, hydration status, and socioeconomic conditions. Finally, the study sample was drawn from a single urban community, which may limit the generalizability of the findings, particularly to rural populations or other regions. Despite these limitations, this study provides valuable insights into the association between renal function markers and depressive symptoms, highlighting the potential relevance of these findings for older adults in China.

Clinically, although the observed effect sizes were modest, routine renal indices may serve as practical markers to flag older adults who could benefit from depression screening during community health check-ups where laboratory infrastructure is available (40). A feasible approach would be to integrate PHQ-9 assessment when Scr or BUN values fall outside age-appropriate reference ranges, with priority given to individuals exhibiting multimorbidity (e.g., diabetes, hypertension) or functional decline.

Future research should employ longitudinal designs incorporating repeated assessments of renal biomarkers (Scr, BUN, cystatin C, and uremic toxin panels) alongside inflammatory markers, while also including direct body-composition measures to disentangle the effects of muscle mass from filtration function. To move beyond association and elucidate the complex interplay among variables, advanced analytical frameworks are crucial. First, pathway analysis or structural equation modeling could be employed to delineate the direct and indirect effects of renal dysfunction on depressive symptoms, and to identify key mediating variables (e.g., inflammatory markers, nutritional status, or specific uremic toxins) along the hypothesized biological pathways (41). Second, applying modern causal inference methodologies—such as marginal structural models to account for time-varying confounding, or propensity score–based analyses—to longitudinal or intervention data would substantially strengthen the causal interpretability of findings regarding the impact of renal function on mental health (42, 43). Mechanistic studies are warranted to determine whether alterations in urea-related pathways—such as carbamylation burden or mTOR signalling activity—correspond to depressive trajectories and cognitive outcomes in ageing. From an analytical perspective, evaluating effect modification by diabetes status and sex, adopting flexible non-linear modelling approaches, and performing robustness analyses (e.g., excluding participants with acute kidney events) would further enhance causal inference and interpretability.

## Conclusion

Among community-dwelling older adults in Guangzhou, higher Scr and BUN were independently associated with greater odds of depressive symptoms, whereas eGFR—modelled as a substitute for Scr—showed a modest inverse association. These findings highlight a clinically meaningful link between renal function and late-life depressive symptoms, underscoring the need for longitudinal studies to establish temporality and causality. Such evidence could ultimately inform scalable, laboratory-based risk stratification and screening approaches for depression prevention in ageing populations.

## Data Availability

The original contributions presented in the study are included in the article/supplementary material. Further inquiries can be directed to the corresponding authors.
